# Attentional and Behavioral Disengagement as Coping Responses to Technostress and Financial Stress: An Experiment Based on Psychophysiological, Perceptual, and Behavioral Data

**DOI:** 10.3389/fnins.2022.883431

**Published:** 2022-07-12

**Authors:** Marion Korosec-Serfaty, René Riedl, Sylvain Sénécal, Pierre-Majorique Léger

**Affiliations:** ^1^Tech3Lab, HEC Montréal, Montréal, QC, Canada; ^2^Digital Business, School of Business and Management, University of Applied Sciences Upper Austria, Steyr, Austria; ^3^Institute of Business Informatics - Information Engineering, Johannes Kepler University Linz, Linz, Austria

**Keywords:** discontinuance, disengagement, technostress, financial stress, techno-unreliability, system response time, digital financial technology, Iowa gambling task (IGT)

## Abstract

Discontinuance of information systems (IS) is a common phenomenon. It is thus critical to understand the decision process and psychophysiological mechanisms that underlie the intention and corresponding behaviors to discontinue IS use, particularly within the digital financial technology usage context, where continuance rates remain low despite increased adoption. Discontinuance has been identified as one coping behavior to avoid stressful situations. However, research has not yet explored this phenomenon toward digital financial technologies. This manuscript builds upon a pilot study that investigated the combined influence of technostress and financial stress on users’ responses toward digital financial decision-making tasks and aims to disentangle the specific impacts of unexpected technology behaviors and perceived financial loss on attentional and behavioral disengagement as coping responses, which may lead to discontinuance from digital financial technology usage. A two-factor within-subject design was developed, where perceived techno-unreliability as variable system response time delays under time pressure and perceived financial loss as negative financial outcomes were manipulated in a 3 × 2 design. Psychophysiological, perceptual, and behavioral data were collected from *N* = 15 participants while performing an adapted version of the Iowa Gambling Task. The results indicate that unexpected technology behaviors have a far greater impact than perceived financial loss on (1) physiological arousal and emotional valence, demonstrated by decreased skin conductance levels and curvilinear emotional valence responses, (2) feedback processing and decision-making, corroborated by curvilinear negative heart rate (BPM) and positive heart rate variability (HRV) responses, decreased skin conductance level (SCL), increased perceptions of system unresponsiveness and techno-unreliability, and mental workload, (3) attentional disengagement supported by curvilinear HRV and decreased SCL, and (4) behavioral disengagement as coping response, represented by curvilinear decision time and increasingly poor financial decision quality. Overall, these results suggest a feedforward and feedback loop of cognitive and affective mechanisms toward attentional and behavioral disengagement, which may lead to a decision of disengagement-discontinuance as a coping outcome in stressful human-computer interaction situations.

## Introduction

Discontinuance of information systems (IS) use is a prevalent phenomenon (Soliman and Rinta-Kahila, 2020). Understanding the decision-making process and psychophysiological mechanisms underlying both the intention to discontinue IS use and actual discontinuing behavior is thus critical, particularly within the digital financial technology usage context, where continuance rates remain low despite increased adoption (e.g., Koghut and Ai-Tabbaa, 2021).

Discontinuance has been identified as one coping behavior to avoid stressful situations (e.g., Beaudry and Pinsonneault, 2005; Britt et al., 2017). However, research had not yet explored this phenomenon in the context of digital financial technology use Korosec-Serfaty et al. (2021a)). A pilot study Korosec-Serfaty et al. (2021b) was thus conducted to investigate the influence of specific factors on two forms of stress within this context: (1) *technostress*, defined as the stress caused by the use of IS and the pervasiveness and expectations of their use in society (Ragu-Nathan et al., 2008; Riedl et al., 2013) and (2) *financial stress*, referred to as the stress caused by financial or economic events (Joo and Grable, 2004). Specifically, in the context of digital financial decision-making, wherein complex yet unreliable IS are used for, paradoxically, reliable outcomes (Butler and Gray, 2006), this pilot study explored the combined effect of two predominant sources of stress (Asebedo and Wilmarth, 2017; Kalischko et al., 2020): (1) *perceived techno-unreliability* due to unexpected technology behaviors caused by delays of system response time (Fischer et al., 2021) and (2) *perceived financial loss*, due to the negative financial outcome of a decision-making task (Joo and Grable, 2004) on users’ psychophysiological and behavioral responses toward digital financial transactions.

Contrary to initial expectations and previous research, which reported increased electrodermal activity (EDA) in response to system response time (SRT) delays (e.g., Trimmel et al., 2003; Riedl and Fischer, 2018) and financial loss (Le et al., 2019), the results from the pilot study showed a decrease in EDA in response to the mixed effect of both forms of stress, indicating lower physiological arousal over time. This result, combined with a reported increase in the general perception of high system unreliability and a measured inferior quality of financial decisions, led to the hypothesis that participants disengaged when performing the financial task as a potential coping response to both technological and financial stress.

Based on these findings, the current research study further explores this relationship by disentangling the influence of unexpected technology behaviors and perceived financial loss on disengagement as a coping response to stress and as a potential antecedent to discontinuance from digital financial technology usage.

### Theory of Stress and Coping

As a lens through which to begin to investigate this relationship, we first draw from the theory of stress and coping (Lazarus and Folkman, 1984), which provides a general framework for understanding psychophysiological and behavioral responses to specific events or stressors, and therefore to perceptions of techno-unreliability and financial loss. This theory models coping responses as a transactional process by combining two interrelated forms of appraisal that continuously influence each other (Beaudry and Pinsonneault, 2005) and often operate in unison (D’Arcy et al., 2014) in response to the subjective interpretation or evaluation of situations. Thus, regardless of its importance, an event may be perceived as stressful by an individual. While in the primary appraisal phase, individuals evaluate whether a situation is benign or stressful, in the secondary appraisal, they assess the nature of the control they may have over the situation and apply a specific strategy to push back or recover from its consequences (D’Arcy et al., 2014). Subsequently, this combination of primary and secondary appraisal results in coping strategies which aim to mitigate the perceived stress.

Research often distinguishes these coping strategies as either problem-focused or emotion-focused (Carver et al., 1989). Problem-focused strategies involve direct and practical efforts to manage or alter the stressful situation and prevail when stressors are appraised as potentially changeable (i.e., high control situations), leading to coping responses such as task adjustment, restraint coping, or seeking social support (Carver et al., 1989). By contrast, emotion-focused strategies involve changing the way one perceives the stressful situation and predominate when the stressor is seen as something that must be accommodated (i.e., low control situations) (D’Arcy et al., 2014). This psycho-affective response drives coping responses of behavioral disengagement, which refers to reducing “one’s effort to deal with the stressor, even giving up the attempt to attain goals with which the stressor is interfering” (Carver et al., 1989: p. 269), or, as posited by this research, attentional disengagement, which refers to one’s effort to redirect attention away from a specific stressor (Wirz and Schwabe, 2020).

**FIGURE 1:**
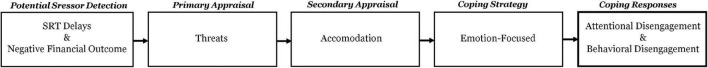
The stress-coping transactional model, applied to digital financial technology usage.

In the context of digital financial technology usage, this research posits that exposure to unexpected technology behaviors as SRT delays and perceived financial loss as negative financial outcome will be assessed as potential threats that must be accommodated. Thus, prompting an emotion-focused coping strategy, resulting in attentional and behavioral disengagement as coping responses (see Figure 1), leading, ultimately to IS discontinuance.

### Information System Discontinuance

Information System discontinuance is a temporal and contextual multifaceted phenomenon, wherein its various antecedents result in distinctly different intentional or behavioral outcomes that occur in different temporal stages of the IS use lifecycle (Soliman and Rinta-Kahila, 2020) and which is influenced by the immediate use context and the nature of IS itself. Soliman and Rinta-Kahila (2020) have identified five forms of discontinuance that encompass the three general IS acceptance and continued use process stages: (1) rejection, which occurs during the exposure phase and is generally based on assumptions; (2) regressive discontinuance, which arises at the acceptance stage, when IS fails to meet users’ initial expectations; (3) temporary discontinuance, which occurs during the continuance phase and where users tend to alternate between periods of vacationing from and returning to IS; (4) replacement which involves a comparison between an IS and its alternative and (5) quitting, which entails breaking a long and stable relationship between the user and the IS, both of which also occur during the continued phase.

Furthermore, there is a basis for these forms of discontinuance as a result of internal coping mechanisms toward IS-related threatening situations (Beaudry and Pinsonneault, 2005). In these situations, users may temporarily discontinue using IS when it becomes a significant source of distraction (York and Turcotte, 2015) as a problem-focused coping strategy. By contrast, when users adopt emotion-focused coping strategies, they may replace an incumbent IS when reaching intense exhaustion (Maier et al., 2015), quit using it when the IS is perceived as intrusive (Chen et al., 2019) or when its use is associated with IS malfunctions (Salo and Frank, 2017). Furthermore, D’Arcy et al. (2014) showed that users mentally disengage from IS when security requirements are perceived as overloading and complex as the consequence of an emotion-focused coping strategy.

This study builds on this last assertion and considers disengagement, and more specifically attentional and behavioral disengagement, as the potential outcome of an emotion-focused coping strategy in response to technology- and financial-related stress and as a potential antecedent to IS discontinuance.

### Operationalizing Disengagement in Response to Technostress and Financial Stress

**FIGURE 2:**
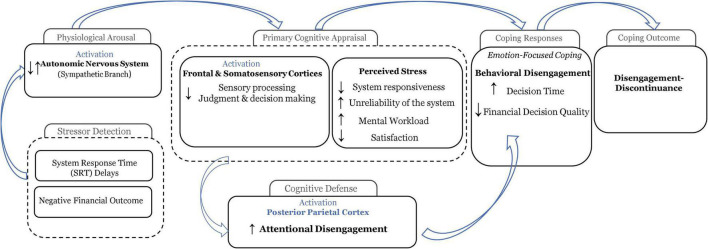
Feedforward-feedback process model of information systems (IS) disengagement toward discontinuance in response to technostress and financial stress.

Building upon the multi-level model of cognitive processing, decision making, and behavior in reaction to stress proposed by Palamarchuk and Vaillancourt (2021), this study posits the following feedforward-feedback process model of IS disengagement as a potential antecedent toward IS discontinuance (see Figure 2). The process model includes the psychophysiological mechanisms that form the basis of attentional and behavioral disengagement as coping responses to unexpected technology behaviors and negative financial outcomes.

The detection of a stressor is first associated with the concurrent activation of the hypothalamic-pituitary-adrenal (HPA) axis and the amygdala, which contributes to the processing of emotional responses, which will then trigger the sympathetic division of the autonomic nervous system (ANS), bringing several physiological changes, including elevation in EDA and electrocardiogram activity (ECG) (Riedl et al., 2012; Korosec-Serfaty et al., 2021a), referred to as physiological arousal. Concurrent with the detection of the stressor and determined by the activation of the autonomic nervous system is the valence of affective states. Negatively valenced affect will be felt when this stressor is assessed as preventing one’s objectives’ achievement (Kemper and Lazarus, 1992). Moreover, valence is highly time-dependent. As such, during and shortly after exposure to the stressor, negative affect is likely to be triggered, yet rapidly followed by positive affect upon withdrawal of the stressor (Giannakakis et al., 2019).

In parallel with the emotional processing of the stressor, neural substrate related to regulatory cognitive appraisal activates the brain areas associated with sensory processing, judgment, and decision-making (Lamm et al., 2007), which then defines the severity of the stressor, affecting the corresponding perceptions, and triggering cognitive defense mechanisms, such as attentional disengagement. Therefore, a change in cognitive processing would be a potential mechanism under which SRT delays and negative financial outcomes influence cognitive appraisal. Negative and delayed feedback have been suggested to alter neurophysiological rhythms and activate both the ventromedial prefrontal cortex, associated with judgment, decision-making and the somatosensory cortex and further associated with sensory-perceptual processing.

Using ECG and EDA activity as proxies for vertical integration of the top-down appraisal brain mechanisms of the ventromedial frontal region (Tranel and Damasio, 1994; Figner and Murphy, 2011; Thayer et al., 2012), previous research on feedback processing found that, in the context of decision-making, heart rate variability (HRV), or the variation of heartbeat over time, was not only higher in response to negative feedback (Vanderhasselt et al., 2015) but also when such feedback was linked to the pressure of a financial decision-making task (Causse et al., 2011). Similarly, skin conductance responses (SCR), the rapid phasic components of electrodermal activity, have been demonstrated to decline after negative feedback following an analogous decision-making task (Brand et al., 2007). Moreover, the timing of the feedback and the factors influencing the nature of the task at hand have been shown to create fluctuations in ECG and EDA activity. Congruent with this, short delayed feedback occurring when conducting a task under time pressure have been reported to stimulate decreased HRV responses while, in the absence of time pressure, short delayed feedback has been demonstrated to increase heart rate beats per minute (BPM) (Boucsein, 2009). By contrast, under time pressure, long-delayed feedback stimulates increases in skin conductance levels (SCL), the background tonic components of EDA (Riedl and Fischer, 2018). These responses indicate that the more individuals tend to be affected by a negative financial outcome or by a delay in system response time, these psychophysiological correlates of feedback processing are activated.

As a parallel process, this unconscious appraisal identifies SRT delays and negative financial outcomes as stressors and potential threats, which then moves appraisal into the conscious realm altering perceptions of system responsiveness, reliability and satisfaction, thus increasing mental workload (Alsuraykh et al., 2019) and creating a continuous feedback loop.

Consequently, we posit that, as an outcome of this unconscious and conscious appraisal, attentional disengagement is activated in a sensory-perceptual feedback loop as a potential defense mechanism. Attentional disengagement has been suggested to alter activity in the posterior parietal cortex, a brain area associated with attention facilitation (Wirz and Schwabe, 2020). Furthermore, previous research found that the combination of increased heart rate and reduced skin conductance, as psychophysiological correlates of attention processing, delineated low task attentional engagement (e.g., Berntson et al., 1993). Moreover, reallocation of attentional resources has been shown to decrease as a consequence of stress (Wirz and Schwabe, 2020). These responses indicate that the more individuals are exposed to stress, the less they can maintain a balance of attentional control, resulting in attentional disengagement. In line with these findings, this study hypothesizes that attentional disengagement will be facilitated in response to SRT and negative financial outcomes.

Subsequently, the combination of these parallel psychophysiological responses, cognitive appraisal, and defense mechanisms leads to the situation being appraised as low-control (i.e., accommodate), resulting in an emotion-focused strategy leading to behavioral disengagement. In line with previous research, this study posits that behavioral disengagement will manifest itself as impaired financial decision quality (ND, 2022) and increased decision time (Cocea and Weibelzahl, 2011) as coping responses (Carver et al., 1989).

Therefore, as the subsequent coping outcome of this psychophysiological process, disengagement-discontinuance will formalize as the intention to discontinue using the digital financial system.

Integrating the theory of stress and coping (Lazarus and Folkman, 1984), the conceptualization of IS discontinuance (Soliman and Rinta-Kahila, 2020), and the feedforward-feedback process model of IS disengagement toward IS discontinuance in response to technostress and financial stress developed previously (see Figure 2), this study aims to answer the following research question: “*What are the specific impacts of unexpected technology behaviors and negative financial outcomes on attentional and behavioral disengagement as coping responses?*” Using a two-factor within-subject experimental design and an adapted version of the Iowa Gambling Task (IGT) (Bechara et al., 2005) to reproduce the context of a financial decision-making task, this study manipulates SRT delays and negative financial outcomes to assess individuals’ responses utilizing psychophysiological measures.

## Materials and Methods

### Participants

Fifteen participants aged 18–45 years (11 females; *M* = 24.7 years; *SD* = 4.7) were recruited for this study. This study focused on members of the general population. Participants were solicited using word-of-mouth, social media and the university student participant panel. The inclusion criteria were: (1) must be over 18 years old, (2) possess normal or corrected to normal vision, and (3) be conversant with computing technologies. The exclusion criteria were: (1) a history of neurological or psychiatric disorders, (2) facial paralysis, and (3) refusal to give informed written consent. The experiment was held in a behavioral laboratory of a major North American university. The total duration of a typical experimental session was 45 min. All subjects gave written informed consent to participate in this study and received $25 as compensation for their participation. This study and all its procedures were carried out in accordance with the Research Ethics Board of the institution where the experiment was conducted (certificate #2021-3910). All participants were informed that they could leave the experiment at any time.

### Experimental Design

**FIGURE 3:**
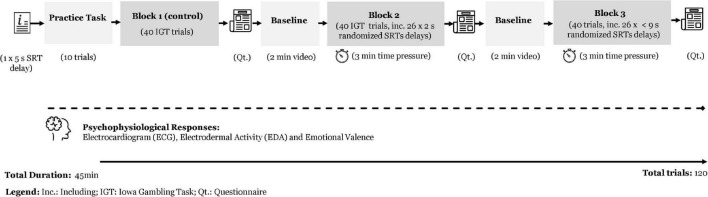
Experimental design.

We utilized a two-factor within-subject experimental design (see Figure 3) to manipulate the antecedents of perceived techno-unreliability and perceived financial loss by inducing SRT delays under time pressure and negative financial outcomes in a 3 [immediate SRT vs. short (2 s) SRT vs. longer (>9 s) SRT] × 2 [negative financial outcome vs. positive financial outcome] design. Time pressure was added to resemble real-life situations and several conditions experienced during human-computer interaction such as those in online environments (Riedl et al., 2013).

#### Financial Decision-Making Task

The experimental task consisted of an adapted computerized version of the Iowa Gambling Task (IGT) (Bechara et al., 1994), a paradigm simulating uncertain and ambiguous decision-making scenarios in real life and used to study neurophysiological mechanisms underlying decisions under stress (e.g., Preston et al., 2007; Simonovic et al., 2018). In this task, participants choose cards from four decks for 100 trials. Each card results in a gain or a loss, whose magnitude and probability are systematically manipulated (Xu and Huang, 2020). Two decks are advantageous as they yield immediate smaller gains and losses, resulting in a net gain, while the other two are disadvantageous as they generate significant monetary gains and occasional significant monetary losses, resulting in a net loss if selected too often. Deck selection is driven by acquired knowledge from trial 50 onward (Preston et al., 2007). After trial 50, a decision is thus considered as made under risk since the contingencies of the task are expected to have been learned (Brand et al., 2007).

In the current study, as shown in Figure 3, the task consisted of 120 trials of the IGT, divided into three consecutive blocks (1, 2, and 3). In each block, participants chose 40 cards from four decks (A, B, C, and D). Similar to the original IGT, Decks A and B were disadvantageous, while Decks C and D were advantageous. Cards were presented face down, with the labels A, B, C, and D below each deck. At the beginning of the task, participants received a virtual amount of $2000 and were instructed to play the game with the objective of maximizing this amount. Their accumulated total amount was displayed at the top right of the screen and updated after each choice. The decks were randomly shuffled at the start of each trial, but the labels always remained left to right. On-click card selection was entirely self-paced, resulting in the positive or negative (i.e., win/loss) financial outcome displayed on the next screen after each card selection.

#### Stress Manipulations

**FIGURE 4:**
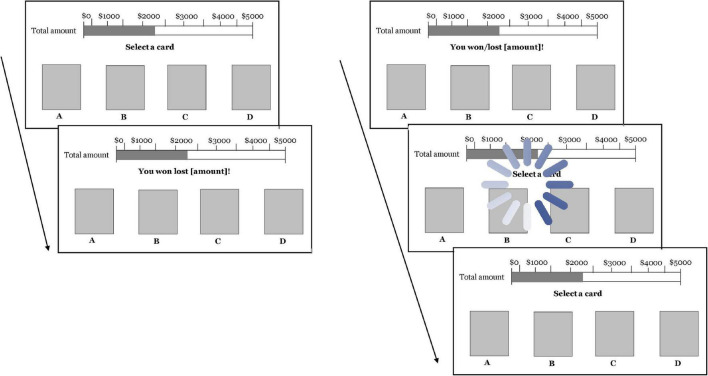
Illustrations of the stress manipulations used in this study. (Left image) Induction of perceived financial loss as a negative financial outcome of the decision-making task. (Right image) Induction of perceived techno-unreliability in the form of system response time (SRT) delays as an animated spinning wheel momentarily preventing participants from carrying on their financial decision.

Each IGT trial’s negative financial outcome (i.e., loss feedback) constituted the financial stressor throughout each block. SRTs delays were induced as an animated spinning wheel appearing on the screen, momentarily preventing participants from making a card selection and carrying on their financial decision (see Figure 4). Before the task began, and to increase the SRTs’ ecological validity, a first 5-s SRT delay interrupted participants while reading the instructions for the task and prior to the ten practice IGT trials. The stress manipulations were counterbalanced by block per participant to avoid ordering effects within the data and ensure internal validity. To induce time pressure, participants were instructed to complete the task within 3 min. This period was chosen after extensive pre-testing. A stopwatch was available to track time with a 3-min countdown. A 2-min video was shown between each block to return participants to a baseline state, as suggested by Boucsein and Thum (1997) and Sharpley et al. (2000).

Illustrations of the stress manipulations used in this study. **(A)** Induction of perceived financial loss as a negative financial outcome of the decision-making task. **(B)** Induction of perceived techno-unreliability in the form of system response time (SRT) delays as an animated spinning wheel momenteraly preventing participants from carrying on their financial decision.

#### Experimental Blocks

Block 1 (control condition) entailed performing 40 trials of the IGT with immediate SRT (i.e., no delays) without time pressure. Block 2 consisted of performing 40 trials of the IGT under time pressure while being randomly interrupted by 26 short (2 s.) SRT delays. Block 3 involved performing 40 trials IGT under time pressure whilst being disrupted by 26 longer (>9 s.; *M* = 10.5; *SD* = 2.12) randomized SRT delays. In all blocks and after each trial, participants were randomly exposed to either positive or negative financial outcomes as a result of their cards selection decision.

### Measures

Electrodermal activity and electrocardiogram (ECG) data were recorded continuously for the duration of the experiment. With well-known foundations, these two biomarkers of autonomic nervous system activation and arousal have been reliably associated with cognitive and affective reactions and responses to stressful situations (e.g., Berntson and Cacioppo, 2007; Braithwaite et al., 2013; Riedl, 2013; Cacioppo et al., 2017). Thus, EDA and ECG were captured as autonomic nervous system measures associated with shifts in emotional arousal to derive SCL, SCR, heart rate (BPM), and heart rate variability (HRV).

As a complement to the measures of psychophysiological arousal, emotional valence was recorded throughout the experiment using automatic facial expression analysis as facial expressions of emotional valence have been shown to signal biological response to stress (Lerner et al., 2007). Automatic facial expression analysis is based on the Facial Action Coding System (FACS) (Ekman and Friesen, 1978) which is used to describe and interpret visually distinguishable facial movements in an anatomically oriented coded system where specific combinations of muscle contractions are associated with certain emotions. The emotional valence ratio, calculated as the intensity of positive emotions subtracted from the intensity of negative expression with the highest intensity, was used to model valence (Höfling et al., 2020).

Throughout the experimental task, decision time and financial decision quality were recorded as measures of performance. Decision time was captured as the total time in each trial that elapsed from the onset of the display of the decks of cards on screen to eventual clicking to select a deck. Financial decision quality was determined as the selection of advantageous decks (i.e., Decks C and D) over the selection of disadvantageous decks (i.e., Decks A and B). A predominance of advantageous decks thus reflected good financial decision quality, whereas a predominance of disadvantageous decks indicated impaired financial decision quality (Garrido-Chaves et al., 2021).

After each experimental block, previously validated self-report scales were used to collect participants’ perceptual data (see Table 1 for detailed measurement items). Perceived system response time was assessed using the corresponding WebQual seven-item subscale (seven-point Likert scale, 1 = strongly disagree to 7 = strongly agree) (Barnes and Vidgen, 2000). Perceived mental workload was measured using the RAW-TLX questionnaire (six dimensions assessed on seven-point Likert scales, 1 = Strongly disagree to 7 = Strongly agree) (Hart and Staveland, 1988; Hart, 2006). Perception of techno-unreliability was assessed using the Digital Stressor Scale corresponding subscale (five-point Likert scale, 1 = Strongly disagree to 5 = Strongly agree) (Fischer et al., 2021). User satisfaction was measured using the one-item scale by Bhattacherjee (2001) (seven-point Likert scale, 1 = Very dissatisfied to 7 = Very satisfied). Perceived system responsiveness and mental workload were measured upon completion of Blocks 1, 2, and 3. Perceptions of techno-unreliability and user satisfaction were assessed after Block 3. All scales items were randomized and used facsimiles to obfuscate their purpose and protect hypotheses, thus preventing rating bias.

**TABLE 1:** Measurement items of self-reports scales.

Variable		Items	Sources
Perceived techno-unreliability	PTU1	I think I was too often confronted with unexpected technology behaviors (e.g., breakdowns or long response time) on this website.	Fischer et al., 2021
	PTU2	I think that I lost too much time due to technical malfunctions.	
	PTU3	I think that I spent too much time trying to fix technical conditions.	
	PTU4	There was just too much of my time wasted coping with the unreliability of this website.	
	PTU5	The hassles with this website (e.g., slow programs or unexpected behaviors) really bothered me.	
Perceived mental workload	PMW1	How mentally demanding was the task?	Hart and Staveland, 1988; Hart, 2006
	PMW2	How physically demanding was the task?	
	PMW3	How hurried or rushed was the pace of the task?	
	PMW4	How successful were you in accomplishing what you were asked to do?	
	PMW5	How hard did you have to work to accomplish your level of performance?	
	PMW6	How insecure, discouraged, irritated, stressed, and annoyed were you?	
Perceived system responsiveness	PSR1	When I used this website there is very little waiting time between my actions and the website’s response	Barnes and Vidgen, 2000
	PSR2	This website loads quickly.	
	PSR3	The website takes long to load.*	
User satisfaction	US1	How do you feel about your overall experience of this website use?	Bhattacherjee, 2001

* Reversed

### Apparatus

The E-Prime 3 software (Psychology Software Tools, Pittsburgh, PA, United States) was used to develop and administer the experimental task. Time markers corresponding to the stimuli presentation were sent by E-Prime to the Observer XT software (Noldus, Wageningen, Netherlands). EDA and ECG data were recorded with a Biopac MP-150 system running *via* the AcqKnowledge 4.4 software (Biopac Systems Inc., Santa Barbara, CA, United States). The Noldus FaceReader software was used to record and model valence (Noldus, Wageningen, Netherlands). *Post hoc* synchronization of the physiological data was run *via* the Cobalt Photobooth software (Courtemanche et al., 2018, 2019, 2022; Léger et al., 2019).

### Analysis

#### Data Pre-processing

Frequency decomposition was applied to the EDA data to derive SCL and SCR. Signal data SCL, SCR, and heart rate responses (BPM and HRV) were then down-sampled at 100 Hz from 256 Hz to produce 1-s averages. HRV ratio was calculated as the low-frequency power divided by high frequency power. FaceReader data were down-sampled at 10 HZ to produce 1-s averages. These data were then segmented by experimental block and by stress manipulations.

#### Data Analyses

Raw data for SCL, SCR, BPM, HRV, and valence were standardized into z-scores at the participant level. Between-block comparison of the main effects of the stress manipulations on SCL, SCR, BPM, HRV, and valence were assessed using linear regression with random intercept (Holm–Bonferroni corrected) after outlier removal (mean ± 3 × SD). Stress manipulation of specific effects were disentangled from main effects, between and within-blocks, using Welch’s *t*-test for unequal sample sizes to maintain Type I error rates close to nominal (Delacre et al., 2017). Due to unreadable data, two participants were excluded from the SCL and SCR analysis.

General perceptions of system responsiveness, techno-unreliability, satisfaction, and subjective workload and its six subscales were averaged per participant and per block. Differences between blocks were assessed with paired sample *t*-tests for equal sample sizes. The strength of the relationships between general perceptions was measured using Pearson correlation after standardizing scales averages into z-scores at the participant level.

Decision time was calculated as the average time elapsed from the final display of the decks (i.e., after the display of the SRT induction in the case of inductions) to clicking on the selected deck. Differences between the specific effects of the stress manipulations between and within blocks were assessed with paired sample *t*-tests.

Financial decision quality was calculated as the average of disadvantageous (A and B) and advantageous (C and D) decks for each block. Specific effects were disentangled from main effects between and within blocks using Fisher’s exact test for categorical data and a small number of observations (Lury and Fisher, 1972).

## Results

### Psychophysiological Responses

#### Heart Rate (BPM)

Comparing between blocks, the analyses of BPM responses (*N* = 15) showed that the BPM averages increased over the duration of the experimental task. As such, the BPM responses were, on average, significantly higher in Block 2 vs. Block 1 [*t*(1738) = 2.63; *p* = 0.02] and in Block 3 vs. Block 1 [*t*(1738) = 2.33; *p* = 0.03]. However, the difference in BPM averages between Blocks 2 and 3 was not significant [*t*(1738) = 1.4; *p* = 0.16].

**FIGURE 5:**
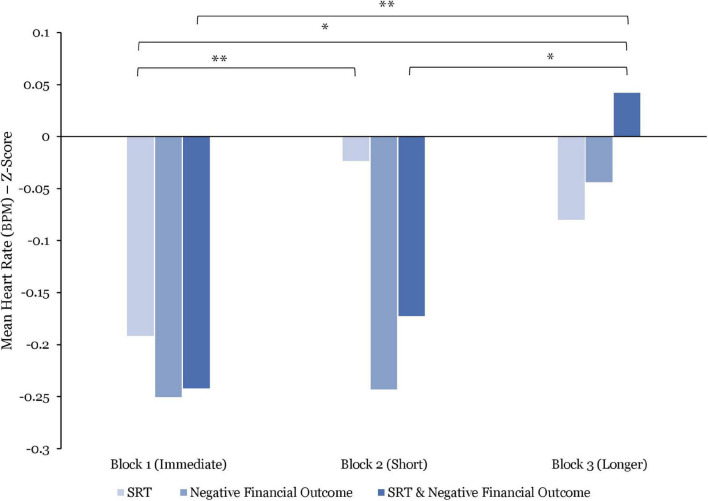
Comparisons of mean heart rate (BPM) responses (z-score) by stress manipulations and by blocks. Significant differences are marked as *p < 0.05; ^**^p < 0.01.

Moreover, comparing the combined effect of SRT and negative financial outcome, the BPM responses were, on average, significantly lower when experiencing immediate SRT and negative financial outcome combined, than while being exposed to longer SRT and negative financial outcome combined [*t*(216) = 2.94; *p* = 0.003; *d* = 0.92]. Similarly, BPM average responses were significantly lower during exposure to short SRT and negative financial outcome combined than while being exposed to longer SRT and negative financial outcome combined [*t*(212) = 2.04; *p* = 0.04, *d* = 0.27]. While the BPM responses were, on average, lower while experiencing immediate SRT and negative financial outcomes combined than during exposure to short SRT and negative financial outcome combined, this difference was found to be statistically non-significant (see Figure 5).

By contrast, when comparing responses to SRT delays (see Figure 5), the BPM averages were significantly higher while being exposed to short SRT vs. experiencing immediate SRT [*t*(597) = 2.91; *p* = 0.003; *d* = 0.22]. Furthermore, while the BPM responses were, on average, lower while being exposed to longer SRT vs. short SRT and to immediate SRT vs. longer SRT, these differences were not significant.

During exposure to negative financial outcomes only, the reported results show that BPM responses were, on average, significantly lower in Block 1 vs. Block 3 [*t*(147) = 2.01; *p* = 0.04; *d* = 0.27]. As shown in Figure 5, average BPM responses to negative financial outcomes only, increased over the course of the experiment. However, these differences were not significant.

The within-block comparison found no significant differences in average BPM responses between SRT and negative financial outcome. However, as illustrated in Figure 5, these data indicate that BPM responses were, on average, higher during exposure to immediate, short and longer SRT vs. negative financial outcome in Block 1, 2, and 3, respectively, suggesting a potential difference that did not reach significance.

#### Heart Rate Variability

With regards to the measure of HRV (*N* = 15), when comparing between blocks, the analysis indicates that the average HRV ratios were significantly higher in Block 1 vs. Block 3 [*t*(1761) = 2.34; *p* = 0.04] and in Block 3 vs. Block 2 [*t*(1761) = 2.42; *p* = 0.04]. The HRV ratios, were, on average, higher in Block 1 vs. Block 2. However, this difference was non-significant.

Furthermore, while these data suggest a decrease in HRV ratios in response to the combined effect of SRT and negative financial outcome over the course of the experiment, the between-block analysis revealed no significant difference.

**FIGURE 6:**
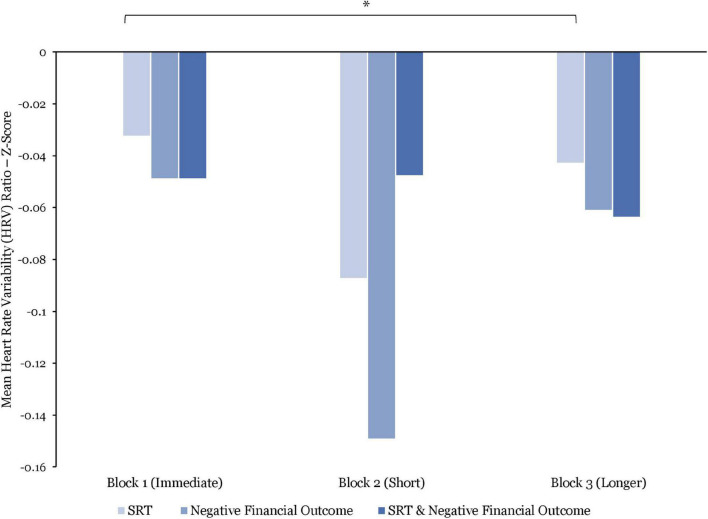
Comparisons of mean heart rate variability (HRV) ratio (z-score) by stress manipulations and by blocks. Significant differences are marked as *p < 0.05.

When assessing HRV responses to SRT only (see Figure 6), the average HRV ratios were significantly higher when exposed to immediate SRT vs. longer SRT [*t*(465) = 2.38; *p* = 0.01; *d* = 0.19]. However, while the HRV ratios were, on average, higher during exposure to immediate SRT vs. short and to longer SRT vs. short SRT, these differences were not significant.

While being exposed to negative financial outcomes only (see Figure 6), the analysis reports that the average HRV ratios were higher in Block 1 vs. Block 2, Block 1 vs. Block 3, and Block 2 vs. Block 3. These differences were found to be non-significant.

Whereas average HRV ratios seem to be higher in response to SRT than to negative financial outcome, the within-block analyses found no significant differences.

#### Skin Conductance Levels

The SCL responses (*N* = 13) strongly declined over the course of the three blocks. As such, the average SCL were significantly lower in Block 2 vs. Block 1 [*t*(1606) = 17.27; *p* < 0.0001], in Block 3 than in Block 2 [*t*(1606) = 12.24; *p* < 0.0001], and in Block 3 vs. Block 1 [*t*(1606) = 17.02; *p* < 0.001].

While these data suggest a variable decrease in the average SCL in response to the combined effect of SRT and negative financial outcome over the course of the experiment, the between-block analyses revealed no significant difference.

**FIGURE 7:**
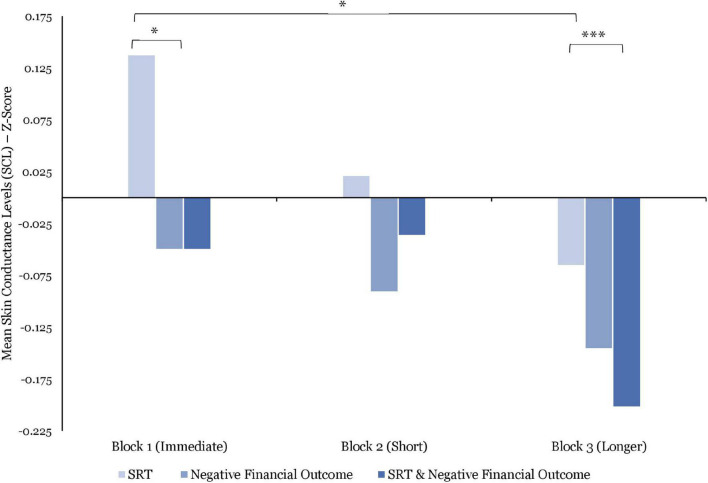
Comparisons of mean skin conductance levels (SCL) (z-score) by stress manipulations and by blocks. Significant differences are marked as *p < 0.05; ^***^p < 0.001.

Comparing between SRT, the SCL averages were significantly lower during exposure to longer SRT vs. immediate SRT [*t*(1500) = 468; *p* = 0.01]. Moreover, while no significant difference was identified between immediate vs. short SRT and short vs. longer SRT, these data point to a strong decrease when moving from immediate, short and longer SRT as shown in Figure 7.

No significant differences were found in the between-block negative financial outcome comparison. However, as illustrated by Figure 7, these data indicate a decline of the average SCL responses over the duration of the experiment.

Comparing within blocks, the SCL responses to immediate SRT were, on average, significantly higher than to negative financial outcomes [*t*(303) = 2.11; *p* = 0.03; *d* = 0.19] as seen in Figure 7. However, although these data present higher SCL average responses when comparing short and longer SRT to negative financial outcomes in Block 2 and 3, respectively, they failed to reach significance. Ultimately, a strong significant difference was found between the exposure to longer SRT only vs. being exposed to longer SRT in combination with negative financial outcome in Block 3 [*t*(278) = 14.01; *p* < 0.001; *d* = 0.13].

#### Skin Conductance Responses

With regards to SCR amplitudes (*N* = 13), the analysis shows an important decrease in their average over the course of the experiment. As such, the SCR amplitudes were, on average, significantly higher in Block 1 vs. Block 2 [*t*(1400) = 2.42; *p* = 0.01], in Block 2 vs. Block 3 [*t*(1400) = 5.69; *p* < 0.001], and in Block 1 vs. Block 3 [*t*(1400) = 5.69; *p* < 0.001].

Results from the comparison of the combined effect of SRT lengths and negative financial outcome show that the SCR amplitudes were, on average, significantly higher after being exposed to immediate SRT and negative financial outcome combined than after exposure to longer SRT and negative financial outcome combined [*t*(155) = 2.89; *p* = 0.004; *d* = 0.41]. However, no further significant differences were found between these combined effects.

**FIGURE 8:**
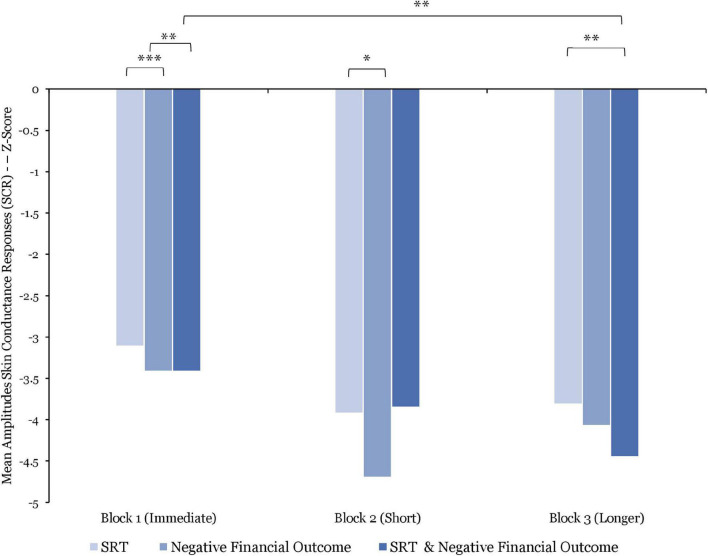
Comparisons of mean amplitude skin conductance responses (SCR) (z-score) by stress manipulations and by blocks. Significant differences are marked as *p < 0.05; ^**^p < 0.01; ^***^p < 0.001.

Comparing between SRT, the data indicate that the SCR amplitudes were, on average, significantly higher after exposure to immediate SRT [*t*(380) = 3.53; *p* < 0.001; *d* = 0.32] vs. short SRT and vs. longer SRT [*t*(393) = 3.2; *p* = 0.001; *d* = 0.29] as illustrated in Figure 8.

After exposure to negative financial outcomes only, the SCR amplitudes were, on average, significantly higher in Block 1 vs. Block 2 [*t*(55) = 2.58; *p* = 0.012; *d* = 0.51]. Whereas no significant differences were identified between Block 2 and 3 and between Block 1 and 3, these data indicate a decline of average SCR amplitudes from Blocks 1 to 3 (see Figure 8).

The within-block comparisons did not reveal any significant differences. However, as shown in Figure 8, the data indicate higher average SCR amplitudes in response to SRT only compared to negative financial outcomes only within each block. These data further show lower average SCR amplitudes in response to the combined effect of SRT and negative financial outcome when compared to response to SRT or negative financial outcome alone, within each block.

### Emotional Valence

**FIGURE 9:**
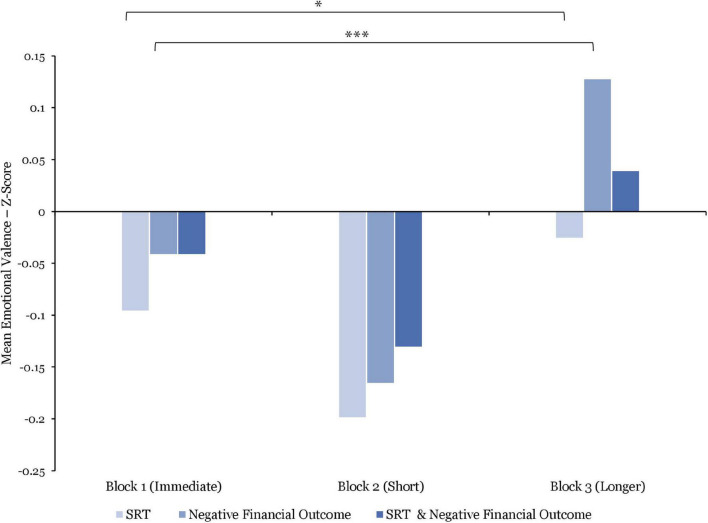
Comparisons of mean emotional valence (z-score) by stress manipulations and by blocks. Significant differences are marked as *p < 0.05; ^***^p < 0.001.

As shown in Figure 9, emotional valence (*N* = 15) was, on average, higher in Block 1 than in Block 2, and significantly lower in Block 2 than in Block 3 [*t*(1736) = 2.99; *p* < 0.01].

Comparing between SRT (see Figure 9), emotional valence responses were, on average, significantly lower during exposure to short vs. longer SRT [*t*(545) = 2.55; *p* = 0.01; *d* = 0.21]. Whereas the average valence responses were at their lowest during exposure to short SRT and at their highest when exposed to longer SRT, no further significant differences were found.

Furthermore, whereas average emotional valence responses were at their lowest when exposed to short SRT only, to negative financial outcome and the combined effect of short SRT and negative outcome in Block 2, they were at their highest while being exposed to longer SRT only, to negative financial outcome only and to the combined effect of longer SRT and negative outcome in Block 3. However, no further significant differences were found between these blocks.

Comparing within blocks, the emotional valence responses to short SRT were, on average, significantly lower than to negative financial outcomes in Block 2 [*t*(123) = 0.49; *p* < 0.001; *d* = 1.47] as seen in Figure 9. In addition, although the results present lower average emotional valence responses when comparing immediate and longer SRT than to negative financial outcomes in Block 1 and 3, respectively, these differences failed to reach significance.

### Performance

#### Decision Time

Comparing between blocks (*N* = 15), the decision time was, on average, significantly longer in Block 1 vs. Block 2 [*t*(1198) = 6.13; *p* < 0.0001] and vs. Block 3 [*t*(1198) = 4.73; *p* < 0.0001]. However, while the average decision time was slightly longer in Block 3 vs. Block 2, this difference was not significant.

**FIGURE 10:**
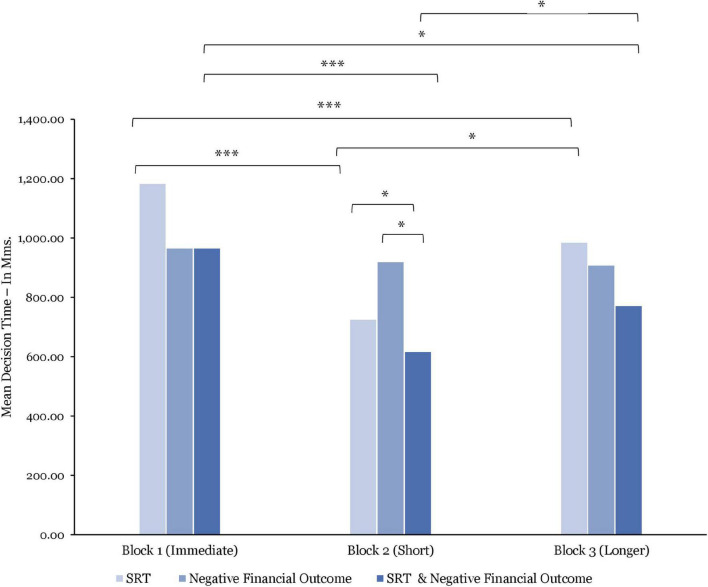
Comparisons of mean decision time by stress manipulations and by blocks. Significant differences are marked as *p < 0.05; ^***^p < 0.001.

As illustrated in Figure 10, the analysis of the combined effect of SRT and negative financial outcome reported that decision time was, on average, significantly longer after being exposed to immediate SRT and negative financial outcome combined than after exposure to short SRT [*t*(266) = 4.78; *p* < 0.001; *d* = 0.52] and longer SRT [*t*(264) = 2.39; *p* = 0.01; *d* = 0.27] in combination with negative financial outcomes. Furthermore, the average decision time in response to the combined effect of short SRT and negative financial outcomes was significantly shorter than to the combined effect of longer SRT and negative financial outcomes [*t*(190) = 2.24; *p* = 0.02; *d* = 0.30].

The results further indicate that the average decision time was significantly longer after exposure to immediate vs. short SRT [*t*(709) = 1.49; *p* < 0.0001; *d* = 0.54] and vs. longer SRT [*t*(698) = 5.10; *p* < 0.0001; *d* = 0.36], and significantly shorter after exposure to short vs. longer SRT [*t*(547) = 2.04; *p* = 0.04; *d* = 0.17].

After being exposed to negative financial outcome only (see Figure 10), the analysis shows that the average decision time was at its highest in Block 1 and at its lowest in Block 3. These between-block differences were, however, found to be non-significant.

Comparing within blocks, the decision time was, on average, significantly longer when experiencing immediate SRT than while being exposed to negative financial outcome in Block 1 [*t*(370) = 2.20; *p* = 0.02; *d* = 0.18] and significantly shorter after being exposed to short SRT than to negative financial outcomes in Block 2 [*t*(64) = 2.01; *p* = 0.04; *d* = 0.30]. Moreover, whereas the data indicate a longer decision time after being exposed to longer SRT compared to negative financial outcomes in Block 2, this difference was not significant.

Ultimately, the decision time was, on average, significantly longer after being exposed to short SRT than to the combined effect of short SRT and negative financial outcome [*t*(300) = 1.97; *p* = 0.04; *d* = 0.18]. By contrast, the average decision time was significantly longer after exposure to negative financial outcome alone than to the combined effect of short SRT and negative financial outcome [*t*(65) = 3.11; *p* = 0.002; *d* = 0.62].

#### Financial Decision Quality

The financial decision quality between-block comparisons (*N* = 15) indicate that the number of disadvantageous decks selected was, on average, significantly higher in Block 1 vs. Block 2 [*t*(1779) = 6.12; *p* < 0.0001], and in Block 1 vs. Block 3 [*t*(1779) = 3.82; *p* < 0.001]. However, the number of disadvantageous decks selected remained significantly higher, on average, than the number of advantageous decks selected, over the full duration of the experimental task [*t*(1779) = 5.11; *p* < 0.0001].

Analyses of the combined effect of SRT and negative financial outcome show that the number of disadvantageous decks was, on average, significantly higher after being exposed to longer SRT and negative financial outcomes combined than after exposure to short SRT and negative financial outcomes combined (*p* < 0.01). Additionally, the average of disadvantageous decks selected as result of the combined effect of short SRT, and negative financial outcome were higher than to the combined effect of longer SRT and negative financial outcome. However, this difference was not significant.

**FIGURE 11:**
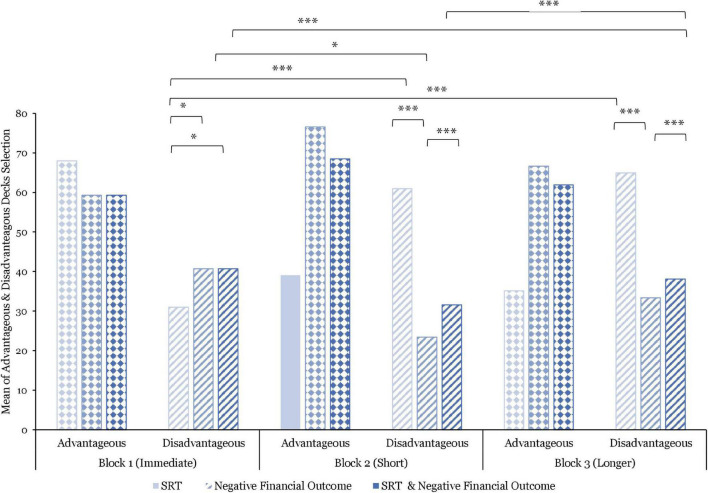
Comparisons of mean advantageous and disadvantageous decks selected by stress manipulations and by blocks. Significant differences are marked as *p < 0.05; ^***^p < 0.001.

As illustrated in Figure 11, the data further indicates that the number of disadvantageous decks selected was, on average, significantly higher after exposure to short than immediate SRT (*p* < 0.00001), and to longer than immediate SRT (*p* < 0.00001). Furthermore, whereas the average number of disadvantageous decks selected was higher after exposure to longer vs. short SRT, this difference was found to be non-significant.

After being exposed to negative financial outcomes only (see Figure 11), the analysis shows that the average of disadvantageous decks selected was significantly lower in Block 2 vs. Block 1 (*p* < 0.05). Further differences between blocks were not significant.

Within-block, the number of disadvantageous decks selected was, on average, significantly lower after being exposed to immediate SRT than to negative financial outcome in Block 1 (*p* < 0.05), to short SRT than to negative financial outcome in Block 2 (*p* < 0.001), and to longer SRT than to negative financial outcome in Block 3 (*p* < 0.001) (see Figure 11).

The average number of disadvantageous decks selected was significantly lower after being exposed to immediate SRT only than to the combined effect of immediate SRT and negative financial outcome (*p* < 0.05). Moreover, the average of disadvantageous decks selected was significantly higher after being exposed to short SRT only than to the combined effect of short SRT and negative financial outcome (*p* < 0.001) and after being exposed to longer SRT only than to the combined effect of longer SRT and negative financial outcomes (*p* < 0.001). Ultimately, no significant differences were found between negative financial outcomes and the combined effects of SRT and negative financial outcome within blocks.

### Perceptual Data

#### Perceptions of System Responsiveness, Techno-Unreliability, and Satisfaction

The overall system responsiveness was perceived, on average, as somewhat unresponsive as reported using the WebQual time perception dimension, measured on a seven-point Likert scale (1 = strongly disagree to 7 = strongly agree) (Barnes and Vidgen, 2000). When comparing between blocks, the system was perceived as significantly less responsive after completing Block 2 vs. Block 1 [*M* = 3.48; *SD* = 1.38; *t*(14) = 9.75; *p* < 0.0001], Block 3 vs. Block 1 [*M* = 4.13; *SD* = 1.64; *t*(14) = 9.75; *p* < 0.0001], and after completing Block 3 vs. Block 2 [*M* = 0.64; *SD* = 0.63; *t*(14) = 3.92; *p* = 0.002]. Thus, participants’ perceptions exactly correspond to the objective SRT manipulations (immediate < short < longer).

After the experimental task, the system used was, on average, perceived as unreliable (*N* = 15) (*M* = 3.9; *SD* = 0.71) as reported using the Fischer et al. (2021) five-point Likert scale (1 = strongly disagree to 5 = strongly agree).

The overall experience was perceived, on average, as somewhat unsatisfactory (*M* = 3.06; *SD* = 1.71) as reported using the Bhattacherjee (2001) seven-point Likert scale (1 = very dissatisfied to 7 = very satisfied).

#### Perception of Mental Workload

The between-blocks comparison indicate a significant difference between the highest perceived workload total score, as reported by the RAW-TLX seven-point Likert scale (1 = very low to 7 = very high), in Block 3 (*M* = 2.63; *SD* = 1.3) and the lowest score in Block 1 (*M* = 2.07; *SD* = 0.8) [*t*(14) = 0.02; *p* = 0.043]. Furthermore, the general perceived workload was considered, on average, as lower in Block 1 vs. Block 2 and in Block 2 vs. Block 3. However, there was no significant difference between these blocks.

Between-blocks statistical differences were found in two dimensions of the subscales: physical demand (i.e., “How physically demanding was the task?”) and frustration (i.e., “How insecure, discouraged, irritated, stressed, and annoyed were you?”). As such, the physical demand scores were, on average, significantly lower in Block 1 vs. Block 3 [*M* = 0.73; *SD* = 0.31; *t*(14) = 2.32; *p* = 0.03] and Block 3 vs. Block 2 [*M* = 0.53; *SD* = 0.21; *t*(14) = 2.47; *p* = 0.02]. Similarly, the average frustration scores were significantly lower in Block 1 vs. Block 3 [*M* = 1.66; *SD* = 0.42; *t*(14) = 3.95; *p* = 0.001] and in Block 2 vs. Block 3 [*M* = 0.73; *SD* = 1.28; *t*(14) = 2.21; *p* = 0.04].

#### Correlations Between Perceptual Measures

When comparing between the perception of techno-unreliability and the different dimensions of the perceived workload as measured by the RAW TLX (see Table 2), negative correlations were found between the general perception of techno-unreliability and the overall performance score [*r*(13) = −0.74; *p* = 0.002]. By contrast, a positive correlation was found between the general perception of techno-unreliability and the general self-reported frustration [*r*(13) = 0.73; *p* = 0.002] scores. A positive correlation was also found with the general perception of temporal demand [*r*(13) = 0.57; *p* = 0.025], which asks the question *How hurried or rushed was the pace of the task?*

**TABLE 2:** Correlations between perceptual data.

TU and the RAW TLX	TU × General perceived workload	*r* = 0.202, *p* = 0.470
	TU × General mental workload	*r* = 0.317, *p* = 0.248
	TU × General physical demand	*r* = 0.280, *p* = 0.310
	TU × General temporal demand	*r* = 0.575, *p* = 0.025*
	TU × General performance	*r* = −0.741, *p* = 0.002**
	TU × General effort	*r* = −0.198, *p* = 0.479
	TU × General frustration	*r* = 0.733, *p* = 0.002**

TU × General system responsiveness		*r* = −0.583, *p* = 0.022*

TU × General satisfaction		*r* = −0.803, *p* < 0.001***

Significant differences are marked *p < 0.05; **p < 0.01; ***p < 0.001.

Moreover, a strong negative correlation was found between the general perceptions of techno-unreliability and system responsiveness [*r*(13) = −0.58; *p* < 0.05.]. Similarly, a strong negative correlation was found between the general perceptions of techno-unreliability and satisfaction [*r*(13) = −0.80; *p* < 0.001].

## Discussion

We posited a feedforward-feedback process model of IS disengagement toward IS discontinuance in response to technostress and financial stress. Overall, the findings provide tentative evidence to support our model.

### Perceptions of System Responsiveness, Techno-Unreliability, and Satisfaction

This feedforward-feedback process model of IS disengagement in response to technostress and financial stress is corroborated from a top-down perspective, starting with the perceptual data, which suggests that the system used for the experimental task was perceived, on average, as significantly and progressively less responsive. As such, the system was perceived as significantly less responsive after exposure to short SRT in Block 2 than immediate SRT in Block 1 (*p* < 0.0001) and after being exposed to longer SRT in Block 3 than to exposure to short SRT in Block 2 (*p* = 0.002). However, while the results were significantly different between blocks, the magnitude of the difference between exposure to short and longer SRTs was less than between the control condition and short SRT. These findings are in line with research that shows the existence of a sensitivity curve associated with SRTs delays. Users typically perceive SRT ≥ 0.2 s (Miller, 1968; Galletta et al., 2006), while if SRTs increase further, they often become less sensitive. SRT delays thus appear to cause some form of distortion of time perception whereby users, for example, tend to perceive an online waiting time of 2 s as being longer than a 10-s waiting time (Chen and Li, 2020). Consequently, the effects of longer SRTs, while still perceived as negative, fall outside of the sensitivity curve and are thus no longer perceived as negatively impacting the task in a linear relationship, potentially due to disengagement (Hirshfield et al., 2014; Kohrs et al., 2016).

After the experimental task, the system used was perceived, on average, as highly unreliable (*M* = 3.9, scale 1–5), and the overall experience as somewhat unsatisfactory (*M* = 3.1, scale 1–7). Significant correlations were found between the general perception of system responsiveness (*p* < 0.05), satisfaction (*p* < 0.001), and techno-unreliability.

### Perception of Mental Workload

With respect to the measurement of perceived mental workload, the results show a significant difference between the lowest score after Block 1 and the highest score after Block 3 (*p* = 0.043). These results can be interpreted as an absence of time pressure and SRT delays in Block 1 vs. time pressure and longer SRT in Block 3. In terms of physical demand, the scores indicate significant differences between Blocks 1 and 3 (*p* = 0.03) and between Blocks 3 and 2 (*p* = 0.02), where the perceived physical demand followed a negative curvilinear pattern from Block 1 to 3. While these results appear to be counterintuitive, a potential explanation may reside in the absence of time pressure and SRT delays in Block 1, followed by the initial exposure to short SRT and time pressure in Block 2, followed by the ultimate exposure to longer SRT and time pressure in Block 3, indicative of a potential disengagement. Similarly supporting these findings, the perceived frustration follows a similar pattern whereby the results indicate significant differences between Blocks 1 and 3 (*p* = 0.001) and between Blocks 3 and 2 (*p* = 0.04). However, in this instance, frustration increased linearly throughout the experiment, further suggesting the intention to disengage with the financial decision task.

An additional indication of potential disengagement may reside in the correlations between the general perception of unreliability of the system, the perceived mental workload score (*p* = 0.002), and the general self-reported frustration score (*p* = 0.002), suggesting that the more unreliable the system became, the less engaged cognitively participants were. Moreover, a correlation was found between the general perception of unreliability of the system and the perceived temporal demand (*p* = 0.025). In this latter case, while the between-block analyses of perceived temporal demand did not indicate significant differences, the pattern of score follows, again, a negative curvilinear pattern, suggesting that the perception of temporal demand increased as the system became unreliable in Block 2 and as the financial decision task became more challenging, with the introduction of time pressure. Subsequently, one may reasonably assume that the introduction of longer SRT with time pressure in Block 3 potentially led participants to disengage with the financial decision task, thus reporting a lower perceived temporal demand.

### Psychophysiological and Behavioral Responses

Taking these results into account, we can now examine the psychophysiological and behavioral measures to disentangle the combined effect of perceived techno-unreliability as specific SRT delays and perceived financial loss as negative financial outcome from their specific effects with regard to disengagement.

#### Combined Effect of System Response Time Delays and Negative Financial Outcome

Concerning the psychophysiological responses to the combined effect of SRT and negative financial outcome, BPM responses increased throughout the experiment. As such, BPM responses were (1) significantly lower when experiencing both immediate SRT and negative financial outcome without time pressure than when exposed to both short SRT and negative financial outcome with time pressure (*p* = 0.003), and (2) significantly lower when experiencing both short SRT and negative financial outcome than when exposed to both longer SRT and negative financial outcome (*p* = 0.04). By contrast, the results show a steady decline in HRV in response to these combined effects. While no significant differences were found between blocks, the observed combined effects of SRT delays and negative financial outcome on BPM and HRV support previous research reporting non-linear relationships between BPM and HRV responses (Goswami et al., 2011). As such, whereas in Block 1, the HRV was relatively high, indicating focused attention (Park et al., 2013), in Block 2, the higher HRV indicates stronger activation of these areas, leading to greater focus, attention, and decision time. During Block 3, however, the HRV ratio declined sharply, potentially signaling attentional disengagement.

Turning to EDA, the data show negative curvilinear SCL responses, whereas differences between blocks were not significant. In combination with the observed effect of increased BPM, these findings may indicate participants’ attentional disengagement toward the performance of the task in response to the combined effect of increased length of SRT delays and negative financial outcome. Additionally, the SCR amplitudes were significantly higher after experiencing immediate SRT and negative financial outcomes combined than after exposure to longer SRT and negative financial outcomes combined (*p* = 0.004). Whereas no other significant differences between blocks were found, these data followed a continual decline over the course of the experiment.

Furthermore, the different emotional valence responses, while non-significant between blocks, followed a curvilinear pattern, potentially indicating that, as the system becomes more unreliable with the introduction of short SRT in Block 2, the emotional valence decreases at this point, only to increase to a more positive level in Block 3 in response to the combined effect of longer SRT and negative financial outcomes. Combined with the observed effect of negative curvilinear SCL responses, these findings may illustrate an asymmetric decoupling providing additional evidence of disengagement whereby, regardless of time pressure, the increase of SRT duration and financial task demand elicit no further affective response.

With regard to the behavioral performance data, the decision time was significantly longer after experiencing the combined effect of immediate SRT and negative financial outcome than to short SRT and negative financial outcomes (*p* < 0.001) and to longer SRT and negative financial outcomes (*p* = 0.01). By contrast, the decision time was significantly shorter when experiencing short SRT and negative financial outcome combined without time pressure than when exposed to longer SRT and negative financial outcome combined with time pressure (*p* = 0.02). Regarding financial decision quality, the data indicated that participants made significantly better financial decisions under time pressure after being exposed to short SRT and negative financial outcome combined than to both longer SRT and negative financial outcome (*p* < 0.01). Further differences between blocks were not significant. However, the data illustrate the same curvilinear pattern observed previously. These findings may provide evidence toward behavioral disengagement in response to the combined effect of longer SRT and negative financial outcome.

When taken together with the previously discussed psychophysiological measures, these last results highlight a pattern in response to the combined effect of SRT delays and negative financial outcome as stressors leading to emotion-focused strategies and attentional disengagement and behavioral disengagement as effective coping responses, illustrating that these coping responses have different psychophysiological and behavioral correlates.

Given these results, it is necessary to investigate which antecedents toward a conclusion of disengagement significantly affect users’ responses.

#### Specific Effects of System Response Time Delays

Concerning the psychophysiological responses to each SRT manipulation type, BPM responses were significantly higher during exposure to short SRT than when experiencing immediate SRT only (*p* = 0.003). Whereas no other significant differences were found, the BPM reported results demonstrated negative curvilinear responses. In addition, similar to Kohrs et al. (2014), longer SRT under time pressure resulted in lower BPM. Moreover, the HRV was significantly higher when experiencing immediate SRT than longer SRT (*p* = 0.01). By contrast with BPM, HRV responses followed a curvilinear pattern, whereby HRV was higher while experiencing immediate SRT with no time pressure, lower during exposure to short SRT, and, again, higher in response to longer SRT under time pressure. These results can be interpreted in terms of attentional focus. As such, during Block 1, participants are in the conditional learning phase of the IGT (Preston et al., 2007), with no perceived time pressure nor SRT delays, thus affording the luxury of time to focus on the financial decision task without any stress inductions. In Block 2, time pressure and random short SRT delays are introduced, resulting in higher mental demands than in Block 1, as highlighted by the self-reported mental workload score and attentional focus. However, in Block 3, under the same time pressure constraints, longer SRT delays are randomly introduced, significantly increasing perceived mental demand (*p* = 0.02) and the need for additional attentional focus under what may have been perceived as impossible conditions, thus leading to potential attentional disengagement evidenced by higher HRV.

With regard to EDA, contrary to previous research that showed increases in SCL during exposure to long SRT with time pressure [for a review, see Boucsein (2009) and Dabrowski and Munson (2011)], SCL responses were significantly lower during exposure to longer SRT under time pressure than while experiencing immediate SRT (*p* = 0.01) with no time pressure. Whereas no further significant differences were found between SRT durations, the data showed a sharp decrease in SCL responses. When combining the results of the SCL responses with the observed effect of HRV as a negative curvilinear pattern, the findings may suggest that, when exposed to longer SRT in Block 3, participants reached a state of effort and attention withdrawal. Furthermore, the amplitudes of the SCR were significantly higher after experiencing immediate SRT than during exposure to short SRT (*p* < 0.001) and to longer SRT (*p* = 0.001). Similar to the previous interpretation, the characteristics of EDA responsivity appear to follow a similar pattern, where SCL decrease over time with a concomitant increase in SCR amplitudes, which may be indicative of a strong negative affective response to both the financial task demands, in terms of time pressure, and increasing SRT delays.

Ultimately, the emotional valence responses were significantly lower while experiencing short SRT under time pressure than during exposure to longer SRT with time pressure (*p* = 0.01). Whereas no further significant differences were found between SRT types, the data followed a positive curvilinear pattern over the course of the experiment, where emotional valence responses were at their lowest during exposure to short SRT delays. These observed effects confirm previous research that showed that short SRT delays of 1.6 s average duration were perceived as aversive (Szameitat et al., 2009). However, in contrast with the majority of previous research on long SRT (Szameitat et al., 2009), our findings suggest that long SRT elicit less negative emotional valence than short SRT. However, these studies did not consider time pressure as a contextual factor, which, in the current study, may have influenced these results. In this case, these results can be interpreted through the combination of strong negative affective responses to exposure to short SRT which is then decoupled during the long SRT, potentially through emotional disengagement from the task at hand.

Investigating performance behavioral data, the reported results showed that the decision time was significantly longer while experiencing immediate SRT than after being exposed to short SRT (*p* < 0.0001) and to longer SRT (*p* < 0.0001). In this case, the positive curvilinear pattern points to the fact that participants’ average decision time was shorter after being exposed to short SRT under time pressure, contrary to previous research that reported higher response time after short delays under time pressure (Szameitat et al., 2009). Ultimately, the positive curvilinear pattern indicates that participants’ average decision time was longer in Block 1 as participants took their time to make their financial decision, shorter in Block 2 when time pressure and short SRT were introduced, and significantly longer again in Block 3 while being exposed to longer SRT, yet still significantly shorter than in Blok 1. With respect to financial decision quality, the data indicate that participants made significantly better decisions at the beginning of the experiment than after being exposed to short (*p* < 0.00001) and longer SRT (*p* < 0.00001). This compares favorably with Szameitat et al. (2009) findings which reported a decline in task performance after exposure to short SRT under time pressure. However, the data further suggest better financial decision quality after being exposed to shorter than to longer SRT. Thus, the financial decision quality deteriorated over the course of the experimental task when comparing between SRT delays only. These findings, coupled with the increase in decision time and HRV ratios in Block 3, suggest that the additional challenges imposed on participants by longer SRT and time pressure resulted from active coping (Ginty et al., 2020), illustrating potential behavioral disengagement.

Taken together, these findings provide evidence pointing toward attentional and behavioral disengagement as an emotion-focused coping response leading to disengagement-discontinuance as a coping outcome in response to unexpected behaviors of technology.

#### Specific Effect of Negative Financial Outcome

With respect to the psychophysiological responses to negative financial outcome only, BPM responses were significantly lower in Block 1 in the absence of time pressure than in Block 3 under time pressure (*p* = 0.04). Whereas no further statistical differences were found between blocks, the data showed a sharp increase in BPM responses from Block 2 to Block 3 under time pressure. Moreover, while no significant differences were found between blocks, HRV followed a positive curvilinear pattern over the duration of the experiment, reaching their lowest in Block 2, and a new high peak in Block 3.

With regards to EDA, no significant differences were found in SCL responses between blocks. However, the reported results demonstrated a progressive decline from Block 1 to Block 3 in response to negative financial feedback. In addition, the magnitude of the SCR to negative financial outcomes only was significantly higher in Block 1 than in Block 2 (*p* = 0.01). Whereas no further significant differences were found between blocks, the reported results followed a positive curvilinear pattern. These findings are similar to those found by Brand et al. (2007), where SCR declined after negative feedback following a financial decision-making task.

Finally, the differences in emotional valence responses, while non-significant between blocks, followed an analogous positive curvilinear pattern than HRV ratios and SCR magnitudes.

Examining behavioral performance-related data in response to negative financial outcomes only, the decision time declined steadily over the course of the experiment. In this case, while differences between blocks were found to be non-significant, the data shows that participants’ average decision time was shorter after a negative financial outcome in Block 1 than in Block 2 and 3. With regard to financial decision quality, the data showed that participants made significantly better decisions after incurring a negative financial outcome in Block 2 than in Block 1 (*p* = 0.03), indicating a potential learning effect. However, while no further significant differences were found between blocks, the quality of the financial decision deteriorated after encountering negative financial outcomes in Block 3. These findings are in line with previous research that showed impaired financial decision quality under time pressure over time (DeDonno and Demaree, 2008).

Other than indicating a potential learning effect of the financial decision task, the lack of significance within these data strongly suggest that perceived techno-unreliability has the stronger effect, whereby the specific effects of negative financial outcomes on psychophysiological and behavioral responses fail to reach significance in comparison.

### Summary

#### Model of Disengagement

The main take-away from the above discussion is that, when disentangling SRT delays from negative financial outcomes, we found that unexpected technology behaviors, and therefore perceived techno-unreliability, has a far greater impact than perceived financial loss on (1) physiological arousal and emotional valence, evidenced by a sharp decrease in SCL and curvilinear valence responses, (2) feedback processing and decision-making under time pressure during the primary appraisal process, evidenced by curvilinear BPM and HRV responses, which were shown to be negative and positive respectively, by decreased SCL, increased perceptions of system unresponsiveness and techno-unreliability, and mental workload, (3) attentional disengagement as a defense mechanism, corroborated by curvilinear HRV and decreased SCL, and (4) behavioral disengagement, denoted by curvilinear decision time and increasingly poor financial decision quality, as coping responses to an emotion-focused coping strategy.

Overall, these results suggest a feedforward and feedback loop of cognitive and affective mechanisms toward disengagement, and more specifically attentional and behavioral disengagement, which can be inferred as leading to a decision of discontinuance as a coping outcome.

To the best of our knowledge, this feedforward and feedback model of cognitive and affective mechanisms is a first attempt to bring together attentional and behavioral disengagement that can be applied to understand disengagement as a holistic process and as a potential antecedent toward IS discontinuance.

#### Decision Dynamics

In terms of decision dynamics, we first argue that exposure to perceived financial stress, in the form of negative financial outcomes, may cause individuals to reassess and thus improve the quality of their financial decisions up to a certain point. While the early stages of exposure to negative financial outcomes may improve financial decision-making by stimulating learning, the repetitive exposure to such financial stressors has an inverse effect. This argument echoes recent findings, which showed a potentially beneficial role of early-stage physiological stress on decision-making and loss aversion (Molins et al., 2021) and adds to the literature by suggesting the existence of a threshold beyond which decision quality and learning deteriorate, further supporting the assertion of behavioral disengagement. Further research is required to identify the exact threshold whereby this beneficial effect of perceived financial stress on decision-making transforms into a negative influence.

Furthermore, we argue that the repeated exposure to unexpected technology behaviors in the form of SRT delays has a cumulative effect on perceived techno-unreliability, and thus a converse effect whereby the quality of financial decision-making deteriorates as both the length of SRT delays and the number of exposures increase. Suggesting therefore that the expected learning effect does not occur over consecutive iterations of the digital financial task and indicating increased attentional disengagement. This is consistent with previous findings, which reported a progressive decline in task performance after exposure to shorter interruptions (2.8 s on average) and longer interruptions (4.4 s on average) (Altmann et al., 2014). Nevertheless, this study extends these findings by suggesting that individuals tend to focus more on the IS and the technology-related aspects of the task rather than on financial decision-making when exposed to repeated, unexpected technology behaviors. This last finding provides support for the assertion that perceived techno-unreliability has a very strong influence toward disengagement in general and behavioral disengagement in particular through a diversion of attentional resources. However, we call for future research incorporating larger sample size to increase observed statistical power and replicate this conclusion.

### Limitations and Future Research

This study comes with a number of limitations that must be acknowledged. First, some of these findings may potentially be the consequence of boredom toward the experimental task as both the latter and attentional disengagement have been shown to share similar physiological signatures (Merrifield and Danckert, 2014). To address this limitation, future research should investigate the neural correlates of attentional disengagement, which has been demonstrated to result in the variation of the N2-posterior-contralateral (N2pc) event-related potential (ERP) component (Luo et al., 2020) to differentiate between attentional disengagement and boredom. Furthermore, by investigating the N2pc in combination with the feedback-related negativity (FRN) ERP component, which has been shown to vary in response to negative and delayed feedback, will provide a more precise picture of the neurophysiological responses to perceptions of techno-unreliability and financial loss.

Another limitation of the current study is that of small sample size, which reduces the positive impact of the results reported here. However, the current sample does provide enough statistical power toward the tentative conclusions provided in the discussion (e.g., Boucsein and Thum, 1997; Garau et al., 2005; Li and Lajoie, 2021). Future research with a larger sample size will add further strength to these results. In addition, individual characteristics, such as computer anxiety, and the intention to discontinue using an IS, were not explicitly measured.

### Contributions and Implications

Notwithstanding these limitations, the present study makes a number of notable contributions to extant knowledge. This study first contributes to the field by answering calls to research to further inquire about the antecedents to IS discontinuance (Soliman and Rinta-Kahila, 2020), investigate users’ coping mechanisms to mitigate the adverse effects of IS (Pirkkalainen and Salo, 2016) and systematically study the relationship between technostress and its psychophysiological and behavioral responses (Riedl and Fischer, 2018).

Moreover, these results contribute to the understanding of IS discontinuance within the context of digital financial technology use, providing novel empirical evidence of disengagement as a potential antecedent to discontinuance. In addition, this study provides a new perspective on emotion-focused coping strategies by suggesting disengagement in general, and attentional and behavioral disengagement in particular, as potential coping responses to perceived techno-unreliability as SRT delays and perceived financial loss due to negative financial outcomes. To this end, we propose a feedforward-feedback process model of IS disengagement-discontinuance in an attempt to explain the processes involved in psychophysiological and behavioral responses to technostress and financial stress. However, this model requires further investigation to validate its predictive quality using, for instance, the previously discussed N2pc and FRN ERP components.

This study further addresses a gap in the literature by disentangling the specific impact of perceptions of techno-unreliability as SRT delays and financial loss as negative financial outcome and showing that perceptions of techno-unreliability have greater impact than perceptions of financial loss in terms of psychophysiological and behavioral responses in the context of a financial decision-making task. More generally, it could potentially be inferred that perceived techno-unreliability, as a ubiquitous form of technostress, affects any decision-making process involving digital technology.

Furthermore, the changes in attention and behavior observed between the short and long SRT delays offer some interesting insights for practitioners. In our experiment, these changes occurred somewhere after 9 s. We suggest that this threshold in attentional and behavioral disengagement is caused by repetitive exposure to unexpected technology behaviors. This finding has implications for IS design in the context of time-sensitive digital financial tasks: under time pressure, short SRT delays (2 s.) have a slight disengaging influence on users’ attention and behaviors. However, longer SRT delays (>9 s; *M* = 10.5) potentially have more serious adverse consequences, as such delays may lead to discontinuance from the system used. Therefore, one could argue that one option to reduce such potential disengagement would be to provide some informative feedback regarding longer SRT delays to ensure that users stay closely engaged (Doherty and Sorenson, 2015).

In addition, previous research in digital retail has shown that user sensitivity to website delays varies across the different stages of their shopping journey, whereby this sensitivity increases at the checkout page compared with the homepage (Gallino et al., 2022). In our experiment, the SRT delay stress manipulations momentarily prevented participants from carrying out their financial decision, thus representing the final step of a digital financial transaction. Our findings thus further imply the importance of reducing such SRT delays within this stage of the digital financial transaction process.

## Conclusion

In conclusion, the primary research question was to identify the specific impacts of unexpected technology behaviors and negative financial loss on attentional and behavioral disengagement as coping responses. Taken together, the findings potentially indicate that perceived techno-unreliability alone has a far greater impact than perceived financial loss, or than perceptions of techno-unreliability and financial loss combined on both forms of disengagement. These findings further suggest a feedforward-feedback loop of cognitive and affective mechanisms toward disengagement, which can be inferred as leading to a decision of discontinuance as a coping outcome.

## Data Availability Statement

The raw data supporting the conclusions of this article will be made available by the authors, without undue reservation.

## Ethics Statement

This study was reviewed and approved by the HEC, Research Ethics Board (REB). This study was carried out in accordance with the recommendations of the Tri-Council Policy Statement: Ethical Conduct for Research Involving Humans, HEC Research Ethics Board (REB) with written informed consent from all subjects. All subjects gave written informed consent in accordance with the Declaration of Helsinki.

## Author Contributions

MK-S: ideation, coordination, design and development, experimental procedures, analysis, interpretation, and writing. RR: ideation, insight, editorial review, and mentorship. SS and P-ML: ideation, insight, editorial review, and supervision. All authors contributed to the article and approved the submitted version.

## Conflict of Interest

The authors declare that the research was conducted in the absence of any commercial or financial relationships that could be construed as a potential conflict of interest.

## Publisher’s Note

All claims expressed in this article are solely those of the authors and do not necessarily represent those of their affiliated organizations, or those of the publisher, the editors and the reviewers. Any product that may be evaluated in this article, or claim that may be made by its manufacturer, is not guaranteed or endorsed by the publisher.
